# What is the best score for predicting difficult laparoscopic cholecystectomy? A diagnostic trial study

**DOI:** 10.1097/JS9.0000000000000354

**Published:** 2023-06-05

**Authors:** Camilo Ramírez-Giraldo, Andrés Isaza-Restrepo, Danny Conde Monroy, Andrea Carolina Castillo-Barbosa, Juan José Rubio-Avilez, Isabella Van-Londoño

**Affiliations:** a Hospital Universitario Mayor—Méderi; b Universidad del Rosario, Bogotá, Colombia

**Keywords:** laparoscopic cholecystectomy, cholecystectomy complications, risk factors, difficult cholecystectomy, sensitivity and specificity

## Abstract

Multiple scores have been created in order to predict difficult cholecystectomy, nonetheless there is not a consensuated standard on which to use. The importance of a predictive score to be able to establish a difficult cholecystectomy would be a relevant instrument in order to better inform the patient, properly call for help when needed, choose the correct staff, and schedule and plan the surgical procedure accordingly.

**Methods:**

A diagnostic trial study was performed. All different predictive scores for difficult cholecystectomy were calculated for each patient. The correlation between the preoperative score and cholecystectomies considered as “difficult” were measured estimating the preoperative score’s predictive value using a receiver operating characteristics curve in order to predict findings for difficult cholecystectomy.

**Results:**

A total of 635 patients between 2014 and 2021 were selected. Selected patients had a mean age of 55.0 (interquartile range: 28.00) and were mostly female (64.25%). Surgical outcomes of patients with difficult cholecystectomy had statistically significant higher rates of subtotal cholecystectomies, drain usage, complications and reinterventions, prolonged surgical times, and longer hospital stay. When analyzing the predictive value on each of the different scores applied, score 4 had the highest performance for predicting difficult cholecystectomy with an area under the curve=0.783 (CI 95% 0.745–0.822).

**Conclusions:**

Difficult cholecystectomies are associated with worse surgical outcomes. The standardization and use of predictive scores for difficult cholecystectomy must be implemented in order to improve surgical outcomes as a result of more meticulous planning when scheduling the procedure.

## Introduction

Highlights

- There is not a standard score for predicting difficult cholecystectomy.
- Multiple risk factors are associated with difficult cholecystectomy.
- Establishing a standard predictive score would improve clinical decision-making.
- Tongyoo *et al.*’s score had the highest performance for predictive value.

Laparoscopic cholecystectomy is one of the most common procedures performed in our population and it is the standard treatment for gallstone disease^1^. Multiple preoperative factors—such as age, sex, body mass index, inflammatory status (leucocytes, neutrophiles, reactive protein C), imaging findings related to cholecystitis (gallbladder wall thickening, impacted gallstones in the gallbladder neck, pericholecystic collection, among others), anatomical variations (Moynihan lump, the presence of more than one cystic artery, among others), history of abdominal surgery, surgeon’s experience and comorbidities such as cirrhosis, symptom duration, time of cholecystectomy (elective, delayed or emergency procedure), among other factors—have been associated with a higher difficulty for the procedure and may imply adverse surgical outcomes^2–4^. It has been proven that it’s unlikely that one sole factor is directly responsible for a difficult cholecystectomy, and it’s more widely considered that the sum of multiple risk factors are associated with difficult cholecystectomy, therefore the importance of a score which may evaluate multiple factors at one given time^3^.

Faced with a grand variety of factors associated with difficult laparoscopic cholecystectomy, multiple studies have designed different predictive scores for difficult cholecystectomy^5,6^ in order to establish an instrument which may better inform the patient, choose appropriate staff, call for help when needed, and plan and schedule the surgical procedure accordingly^6^. Nonetheless, there is not a generalized consensus on the usage of the scores described nor a comparison between their utility. As a result, the objective of this study is to evaluate the predictive value for each of the different scores available in order to better predict difficult laparoscopic cholecystectomy.

## Patients and methods

A diagnostic trial study was performed to evaluate scores to predict difficult laparoscopic cholecystectomy. These cholecystectomies were performed by a group of 35 surgeons with variable experience that ranged from 4 to 30 years, out of which each and every surgeon performs around 60 cholecystectomies a year on average. A simple random sampling was done until the calculated sample size was reached. Different variables were collected on an anonymous database. This study was reviewed and approved by the Universidad del Rosario (number DVO005 2096-CV1613) ethics committee. We followed STARD guidelines in order to report this study^7^.

### Patients

Patients under 18 years of age, patients with scheduled open cholecystectomy, patients with diagnosed gallbladder cancer, patients with cholecystectomy associated to other procedures (laparoscopic common bile duct exploration, gastrectomy, pancreatoduodenectomy, among others) and patients whose registry did not include variables of interest were excluded from the study.

Laparoscopic cholecystectomy indications included all cases in which the main indication was biliary cholic, pancreatitis, choledocholithiasis, cholecystitis or a combination of these; and in all cases there was at least one diagnostic image which confirmed biliary disease. Patients diagnosed with cholecystitis were classified according to their severity and treatment was established using Tokyo guidelines^8,9^. Additionally, ASGE criteria for risk of choledocholithiasis was established in order to define a management plan; cholecystectomy with no additional studies was defined for the low-risk group, magnetic resonance cholangiography for the intermediate-risk group and endoscopic retrograde cholangiopancreatography (ERCP) for the high-risk group^10^. In cases with diagnosis of pancreatitis cholecystectomy was performed once pancreatitis was resolved.

### Study design

A literature review was performed in order to find predictive scores for difficult cholecystectomy in which only preoperative variables were included, findings are reported in Table 1. Every patient had their preoperative score measured using each of the predictive scores included for difficult cholecystectomy.

**Table 1:** Scores found in literature for predicting difficult cholecystectomy.

Score	Author	Variables		Points	AUC
Score 1	Gupta *et al*.^11^Randhawa *et al*.^12^Tongyoo *et al*.^13^Agrawal *et al*.^14^	Age (years)	≤50>50	01	0.860.820.750.87
		Sex	MaleFemale	10	
		History of hospitalization for acute cholecystitis	NoYes	04	
		BMI	<25 kg/m^2^25–27.5 kg/m^2^>27.5 kg/m^2^	012	
		Abdominal scar	NoInfraumbilicalSupraumbilical	012	
		Palpable gallbladder	YesNo	10	
		Gallbladder wall thickness	<4 mm≥4 mm	02	
		Pericholecystic collection	NoYes	01	
		Impacted stone	NoYes	01	
Score 2	Siddiqui *et al*.^15^	Gallbladder wall thickness	<4 mm≥ 4 mm	02	NR
		Transverse diameter of gallbladder	<5 cm≥5 cm	02	
		Presence of impacted stones	NoYes	02	
		Common bile duct diameter	≤6 mm>6 mm	02	
		Presence of pericholecystic collection	NoYes	010	
		No. stones >1	NoYes	10	
		Liver size ≥ 15.5 cm	NoYes	1	
Score 3	Kama *et al*.^16^Bulbuller *et al*.^17^	Sex	MaleFemale	110	0.83NR
		Abdominal tenderness	NoYes	09	
		Previous upper abdominal operation	NoYes	08	
		Thickened gallbladder wall	NoYes	013	
		Age (years)	≥60<60	50	
		Acute cholecystitis	NoYes	015	
		Constant		−20	
Score 4	Tongyoo *et al*.^13^Tongyoo *et al*.^18^	Age (years)	≤50>50	01	0.820.80
		Sex	MaleFemale	10	
		History of previous biliary inflammation and procedure (previous acute cholecystitis, cholangitis, ERCP)	NoYes	04	
		BMI	<25 kg/m^2^25–27.5 kg/m^2^>27.5 kg/m^2^	012	
		Abdominal scar	NoInfraumbilicalSupraumbilical	012	
		Contracted gallbladder	YesNo	10	
		Gallbladder wall thickness	<4 mm≥4 mm	02	
		Pericholecystic collection	NoYes	01	
		Impacted gallstone	NoYes	01	
Score 5	Carrizo *et al*.^19^	Age	≤60 years>60 years	02	NR
		Sex	MaleFemale	1.50	
		BMI	≤30 kg/m^2^> 30 kg/m^2^	01	
		Previous surgery (upper hemiabdomen)	NoYes	02	
		Gallbladder wall thickness	≤3 mm>3 mm	02	
		Common bile duct stone	NoYes	08	
		Leucocytes	≤10.0×10^3^>10.0×10^3^	02	
Score 6	Nassar *et al*.^6^Ramírez-Giraldo *et al*.^20^	Age	<40 years≥40 years	01	0.780.88
		Gender	MaleFemale	10	
		ASA classification	IIIIIIIV–V	0127	
		Primary diagnosis	PancreatitisBiliary colicCBD stoneCholecystitis	0014	
		Gallbladder wall thickness	<3 mm≥3 mm	02	
		Common bile duct diameter	≤6 mm>6 mm	01	
		Preoperative ERCP	NoYes	01	
		Admission type	ElectiveDelayEmergency	012	
Score 7	Alponat *et al*.^21^	Acute cholecystitis	NoYes	01.1390	NR
		Gallbladder wall thickness	<3.5 mm≥3.5 mm	01.3227	
		Leucocytes	≤11.0×10^3^>11.0×10^3^	01.3063	
		Elevated alkaline phosphatase	NoYes	00.8014	
		Constant		−4.2149	

ASA, ASA Physical status classification system; AUC, area under the curve; ERCP, endoscopic retrograde cholangiopancreatography; NR, not reported.

Scores containing variables not routinely measured on patients scheduled for laparoscopic cholecystectomy in our institution were excluded from the study, such as one by Bourgouin and colleagues which measured fibrinogen, another one by Lipman and colleagues which measured albumin, one score by Schrenk and colleagues which included the inability to observe the gallbladder in intraoperative cholangiography as one of its variables and a score described by Vivek, and colleagues which is difficult to calculate due to the amount of variables and the inclusion of intraoperative variables^22–25^.

There are multiple other studies in which risk factors have been evaluated; however, none of these studies designed scores from identifiable factors for predicting difficult cholecystectomy, and as a result were excluded from the study^26–34^.

We consider as difficult cholecystectomy (reference standard) the presence of at least one of the following conditions in an intraoperative setting: bile duct injury, non-evident anatomical visualization, Mirizzi syndrome, severe inflammation of the Calot triangle, conversion to laparotomy, scleroatrophic gallbladder or pericholecystic abscess. This was defined by a panel of experts with a majority consensus of greater than or equal to 80% as the presence of any one of these findings^35^.

The surgeon that would perform the cholecystectomy was not aware of any of the preoperative score results as these were calculated by the research team in a retrospective manner.

### Statistical analysis

Multiple samples were calculated according to the sensibility and specificity for each of the scores (Table 1), out of which the biggest sample size was chosen. The biggest sample size was calculated with a 78% sensibility and 72% specificity as was reported by Kama *et al*.^16^ while the prevalence of difficult cholecystectomy previously reported in our population is 51.72%^20^.

Data distribution was evaluated using the Shapiro–Wilk and Kolmogorov–Smirnov test, yielding a non-normal distribution. A description of demographic, clinic, paraclinical, surgical and outcome variables was made using the following parameters: categorical variables were described as ratios and continuous variables as a mean average with a respective interquartile range. A bivariate analysis was performed with a χ^2^ value on categorical variables and with the Mann–Whitney test on continuous variables in order to compare differences between the variables in respect to difficulty (easy vs. difficult), considering a statistically significant *P* less than 0.05.

We calculated the score for every different predictive score for difficult cholecystectomy on each patient (Table 1). The correlation between the preoperative score and cholecystectomies considered as difficult was gauged by means of a receiver operating characteristic curve estimating the preoperative score’s predictive value to acknowledge findings defined as difficult cholecystectomy. The area under the curve (AUC) for each of the different predictive scores for difficult cholecystectomy were compared in order to assess if there were statistically significant differences between them.

The entirety of this analysis was executed on SPSS26, deeming a *P* less than 0.05 as statistically significant.

## Results

**Figure 1:**
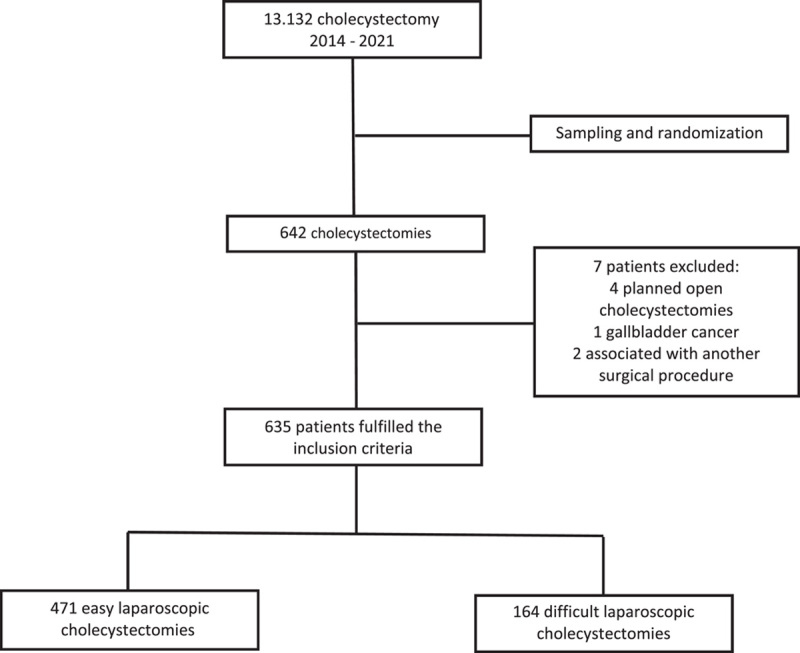
Study selection process flowchart.

Between January 2014 and December 2021, 13 132 cholecystectomies were performed in our institution. A total sum of 635 patients were included in this study. Four hundred seventy-one were classified as easy laparoscopic cholecystectomies (74.17%) and 164 were classified as difficult (25.83%). The selection process is shown in the following flowchart (Fig. 1).

Selected patients had a mean age of 55.0 (interquartile range: 28.0) years and were predominantly female (64.25%). In Table 2 other demographic, clinic and paraclinical aspects in cholecystectomized patients are reported with the difference between these according to cholecystectomy difficulty.

**Table 2:** Demographic, clinical and surgical characteristics by difficulty.

	*N* (%)	Easy (%) *n*=471	Difficult (%) *n*=164	*P*
Age (median) (IQR) years	55.00 (28.00)	51.00 (27.00)	61.5 (23.25)	**<0.001** *
Sex				**<0.001**
Female	408 (64.25)	329 (69.85)	79 (48.17)	
Male	227 (35.75)	142 (30.15)	85 (51.83)	
Body mass index	26.40 (5.35)	26.3 (5.309	26.70 (5.30)	0.375*
ASA classification				**0.011**
1	254 (40.00)	192 (40.76)	62 (37.80)	
2	274 (43.15)	213 (45.22)	61 (37.20)	
3	103 (16.22)	63 (13.38)	40 (24.39)	
4–5	4 (0.63)	3 (0.64)	1 (0.61)	
Comorbidities				
Diabetes mellitus	74 (11.65)	48 (10.19)	26 (15.85)	0.052
Arterial hypertension	171 (29.93)	121 (25.69)	50 (30.49)	0.233
Chronic obstructive pulmonary disease	17 (2.68)	11 (2.34)	6 (3.66)	0.366
Chronic kidney disease	11 (1.73)	7 (1.49)	4 (2.44)	0.421
Cardiovascular disease	44 (6.93)	27 (5.73)	17 (10.37)	**0.044**
Liver disease	7 (1.19)	6 (1.27)	1 (0.61)	0.483
History of abdominal surgery				
Supraumbilical	28 (4.41)	19 (4.03)	9 (5.49)	0.435
Infraumbilical	223 (35.12)	173 (36.73)	50 (30.49)	0.149
Previous episodes of cholecystitis	6 (0.94)	5 (1.06)	1 (0.61)	0.606
Abdominal pain	513 (80.79)	363	150 (91.46)	**<0.001**
Palpable gallbladder	8 (1.26)	1 (0.21)	7 (4.27)	**<0.001**
Charlson comorbidity index (median) (IQR) points	1.00 (2.00)	1.00 (2.00)	2.00 (3.00)	**<0.001** *
Anticoagulant agents	12 (1.89)	7 (1.49)	5 (3.05)	0.206
Antiplatelet agents	53 (8.35)	33 (7.01)	20 (12.20)	**0.039**
Lab tests (median) (IQR)				
Leucocytes (×10^3^)	9.85 (5.59)	9.31 (5.01)	11.95 (5.66)	**<0.001** *
Haemoglobin (mg/dl)	14.90 (1.90)	14.80 (1.70)	15.00 (2.00)	**0.008** *
Total bilirubin (mg/dl)	0.76 (0.98)	0.71 (0.91)	0.96 (1.24)	0.108*
Alkaline phosphatase (mg/dl)	105.00 (88.00)	105.00 (89.00)	105.50 (87.25)	0.930*
Aspartate aminotransferase (mg/dl)	27.00 (74.75)	27.00 (95.25)	28.00 (39.25)	0.587*
Alanine aminotransferase (mg/dl)	31.00 (104.00)	30.1 (142.50)	34.00 (69.00)	0.938*
Image findings				
Impacted stone	40 (6.30)	21 (4.46)	19 (11.59)	**0.001**
Pericholecystic collection	81 (12.76)	35 (7.43)	46 (28.05)	**<0.001**
More than one stone	620 (97.64)	461 (97.88)	159 (96.95)	0.501
Scleroatrophic	6 (0.94)	6 (1.27)	0 (0.00)	0.146
Gangrenous/perforated	24 (3.78)	1 (0.21)	23 (14.02)	**<0.001**
Liver >15.5 cm	12 (1.89)	9 (1.91)	3 (1.83)	0.947
Gallbladder wall thickness	2.00 (2.00)	2.00 (2.00)	4.00 (2.00)	**<0.001** *
Gallbladder transverse diameter (median) (IQR) cm	3 (0.00)	3 (0.00)	3 (0.00)	0.733*
Bile duct diameter (median) (IQR) mm	3 (0.00)	3 (0.00)	3 (0.00)	0.382*
Primary diagnosis				
Biliary cholic	204 (32.13)	173 (36.73)	31 (18.90)	**<0.001**
Pancreatitis	53 (8.35)	41 (8.70)	12 (7.32)	0.580
Choledocholithiasis	74 (11.65)	48 (10.19)	26 (15.85)	0.052
Cholecystitis	272 (42.83)	150 (31.85)	122 (74.39)	**<0.001**
Tokyo classification				**<0.001**
I	92 (14.49)	69 (14.65)	23 (14.02)	
II	104 (16.8)	59 (12.53)	45 (27.44)	
III	76 (11.97)	22 (4.67)	54 (32.93)	
Preoperative ERCP				**0.001**
No	554 (87.24)	423 (89.81)	131 (81.10)	
Yes	81 (12.76)	48 (10.19)	33 (18.90)	
Type of admission				**<0.001**
Elective	122 (19.21)	108 (22.93)	14 (8.54)	
Delayed	507 (79.84)	362 (76.86)	145 (88.41)	
Emergency	6 (0.94)	1 (0.21)	5 (3.05)	
Previous cholecystostomy	3 (0.47)	2 (0.42)	1 (0.61)	0.766
Time from admission to surgical procedure (median)(IQR) days	3.00 (4.00)	3.00 (4.00)	3.00 (4.00)	0.175

*P* values were obtained from the χ^2^ test.

Values in bold indicate statistically significant *P* values (*P*<0.05).

ASA, ASA Physical status classification system; ERCP, endoscopic retrograde cholangiopancreatography; IQR, interquartile range.

* *P* values were obtained from the Mann–Whitney test.

Surgical outcomes evidenced a statistically significant higher ratio of subtotal cholecystectomies, drain usage, complications and reinterventions, prolonged surgical times and longer hospital stay in the group of patients classified as difficult cholecystectomies. There were also higher mortality rates in the difficult cholecystectomies group; however, the difference was not statistically significant (Table 3).

**Table 3:** Postoperative cholecystectomy surgical outcomes according to difficulty.

	*N* (%)	Easy (%) *n*=471	Difficult (%) *n*=164	*P*
Type of cholecystectomy				**<0.001**
Total	610 (96.06)	469 (99.58)	141 (85.98)	
Subtotal	25 (3.94)	2 (0.42)	23 (14.02)	
Conversion rate				**<0.001**
No	616 (97.01)	471 (100.00)	145 (88.41)	
Yes	19 (2.99)	0	19 (11.59)	
Drain usage				**<0.001**
No	601 (94.65)	467 (99.15)	134 (81.71)	
Yes	34 (5.35)	4 (0.85)	30 (18.29)	
Surgical time (median) (IQR) min	60.00 (10.00)	60.00 (2.00)	60.00 (60.00)	**<0.001** ^*^
Hospital stay (median) (IQR) days	3.00 (4.00)	3.00 (3.00)	5.00 (5.00)	**<0.001** ^*^
Complications				
Biliary leak	2 (0.31)	0	2 (1.22)	**0.016**
Bile duct injury	1 (0.16)	0	1 (0.61)	0.090
Intestinal injury	2 (0.31)	1 (0.21)	1 (0.61)	0.434
Surgical site infection	13 (2.05)	7 (1.49)	6 (3.66)	0.091
Perioperative AMI	1 (0.16)	1 (0.21)	0 (0.00)	0.555
Healthcare-associated pneumonia	2 (0.31)	1 (0.21)	1 (0.61)	0.434
Healthcare-associated urinary tract infection	1 (0.16)	1 (0.21)	0 (0.00)	0.555
Pleural effusion	4 (0.63)	2 (0.42)	2 (1.22)	0.268
Reintervention				**<0.001**
No	625 (98.43)	469 (99.58)	156 (95.12)	
Yes	10 (1.57)	2 (0.42)	8 (4.88)	
Clavien–Dindo				**<0.001**
I	22 (3.46)	11 (2.34)	11 (6.71)	
II	12 (1.89)	4 (0.85)	8 (4.88)	
IIIA	12 (1.89)	5 (1.06)	7 (4.27)	
IIIB	6 (0.94)	2 (0.42)	4 (2.44)	
IV	1 (0.16)	0	1 (0.61)	
V	5 (0.79)	2 (0.42)	3 (1.83)	
Mortality				0.080
No	630 (99.21)	469 (99.58)	161 (98.17)	
Yes	5 (0.79)	2 (0.42)	3 (1.83)	

*P* values were obtained from χ^2^ test.

Values in bold indicate statistically significant *P* values (*P*<0.05).

AMI, acute myocardial infarction; IQR, interquartile range.

* *P* values were obtained from Mann–Whitney test.

**Figure 2:**
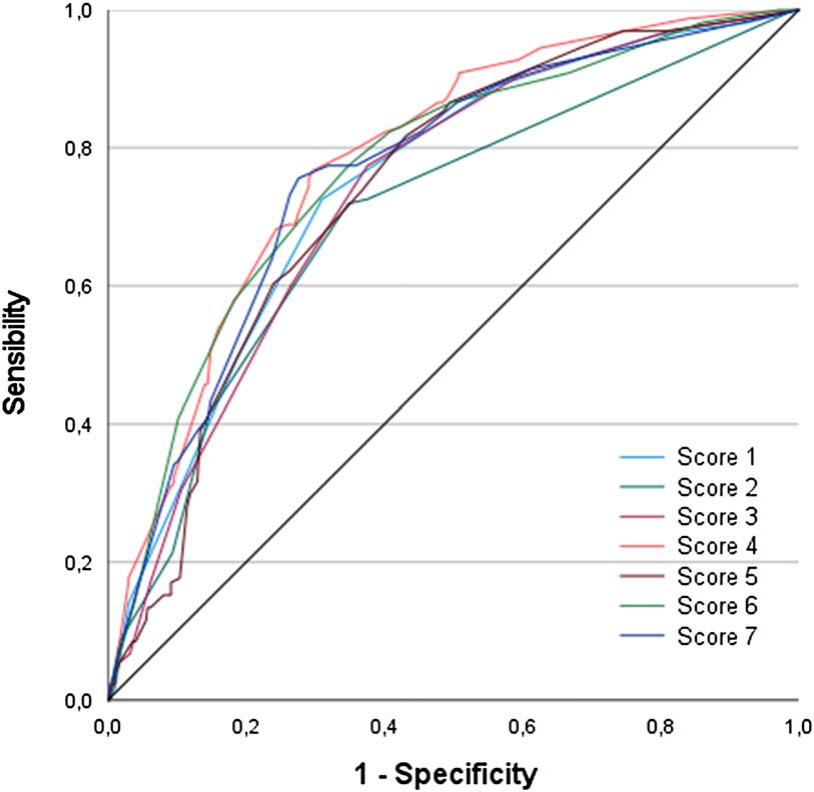
ROC Receiver curves for scores evaluated.

When measuring predictive capacity for difficult cholecystectomy with each score we found score 4, described by Tongyoo *et al*.^13^ , as the score with better performance for predicting a difficult cholecystectomy. On the other hand, score 2 which focused exclusively on image findings, reported by Siddiqui *et al*.^15^ was the one with the lowest predictive performance (Fig. 2 and Table 4).

**Table 4:** AUC for each score.

Score	AUC	CI 95%
Randhawa (score 1)	0.747	0.704–0.789
Siddiqui (score 2)	0.703	0.655–0.750
Kama (score 3)	0.736	0.694–0.778
Tongyoo (score 4)	0.783	0.745–0.822
Carrizo (score 5)	0.740	0.699–0.781
Nassar (score 6)	0.768	0.726–0.809
Alponat (score 7)	0.761	0.719–0.803

AUC, area under the curve.

Finally, we made a comparison between each score’s AUC, in which we can evidence score 4, which had the best performance, was statistically significantly superior to scores 1, 2, 3 and 5. And despite score 4 having a superior value to scores 6 and 7, there was not a statistically significant difference between them (Table 5).

**Table 5:** AUC differences between each score.

	AUC difference	*P*
Randhawa (score 1)–Siddiqui (score 2)	0.044	**0.018**
Randhawa (score 1)–Kama (score 3)	0.011	0.383
Randhawa (score 1)–Tongyoo (score 4)	0.037	**0.022**
Randhawa (score 1)–Carrizo (score 5)	0.007	0.755
Randhawa (score 1)–Nassar (score 6)	0.021	0.269
Randhawa (score 1)–Alponat (score 7)	0.015	0.438
Siddiqui (score 2)–Kama (score 3)	0.033	0.114
Siddiqui (score 2)–Tongyoo (score 4)	0.081	**<0.001**
Siddiqui (score 2)–Carrizo (score 5)	0.038	0.127
Siddiqui (score 2)–Nassar (score 6)	0.065	**0.001**
Siddiqui (score 2)–Alponat (score 7)	0.059	**0.001**
Kama (score 3)–Tongyoo (score 4)	0.047	**0.010**
Kama (score 3)–Carrizo (score 5)	0.004	0.800
Kama (score 3)–Nassar (score 6)	0.032	0.088
Kama (score 3)–Alponat (score 7)	0.026	0.222
Tongyoo (score 4)–Carrizo (score 5)	0.043	**0.017**
Tongyoo (score 4)–Nassar (score 6)	0.016	0.121
Tongyoo (score 4)–Alponat (score 7)	0.022	0.100
Carrizo (score 5)–Nassar (score 6)	0.028	0.138
Carrizo (score 5)–Alponat (score 7)	0.021	0.274
Nassar (score 6)–Alponat (score 7)	0.006	0.668

Measured by a non-parametric estimate.

Values in bold indicate statistically significant *P* values (*P*<0.05).

AUC, area under the curve.

## Discussion

This study identified that cases considered as difficult cholecystectomy are associated to higher rates of subtotal cholecystectomy, complications and reinterventions, thus the importance in being able to predict difficult cholecystectomy. Out of the 7 scores for predicting a difficult cholecystectomy evaluated, score 4 (AUC 0.783) had the highest performance. This scale is based on a scale designed by Randhawa (score 1) in which the criteria “palpable gallbladder” was modified for “contracted gallbladder” and “history of hospitalization for acute cholecystitis” was modified to “history of previous biliary inflammation and procedure (previous acute cholecystitis, cholangitis, ERCP)”. The scores reported by Nassar and Alponat had lower performances compared with score 4 but the difference was not statistically significant^6,13,21^.

That preoperative identification of difficult cholecystectomy may imply a greater surgical risk has been a relevant topic of discussion in recent years. Risk assessment before surgical intervention allows for better preparation for the procedure, a more accurate measurement of surgical times, adequate planning for different services not available 24 h (availability of a hepatobiliary surgeon, intraoperative imaging, among others), and counselling and risk breakdown are more proper and realistic for the patient when signing informed consent^14^.

Traditionally, the approach to difficult cholecystectomy differs depending on the surgeon. The wide array of surgical techniques available are mainly based on surgeon experience and different strategies have been devised to guide professionals on an individualized management^36^. On the other hand, the term “difficult” is not standardized; some authors define difficulty on surgical time while others determine it as the need of conversion to open procedure and further intraoperative findings; and as a result, each of the scores yield to different reference standards^27,36,37^. In this review, in order to establish a reference point on the definition of difficult cholecystectomy we employed a consensus recently published by an expert surgeon panel in Spain which considers clinical aspects, imaging features and intraoperative findings^35^. These multiple definitions for difficult cholecystectomy limit the implementation of a specific score to predict difficulty and act as a barrier when trying to reach a unanimous standard.

Out of multiple scores created for the assessment of difficult cholecystectomy, few have been frequently used in a clinical setting due to the complexity and difficult applicability of some variables^38^. Even though Vivek *et al.* ^24^ published a score with a high AUC (0.956), the 22 variables required to adequately measure it (including an intraoperative variable) pose a challenge when trying to assess difficulty on an everyday basis. Moreover, other scores consider variables which are not routinely measured and are difficult to obtain, such as preoperative fibrinogen and intraoperative cholangiography^22,23^.

In our review aspects related to difficult cholecystectomy included age, male sex, ASA Physical status classification system, antiplatelet agents, history of cardiovascular disease, palpable gallbladder, leukocytosis, image findings consistent with cholecystitis, pericholecystic collection, gangrenous or perforated gallbladder, impacted stone, patients with Tokyo III score and urgent surgery; variables which were included in various scores. Previous ERCP was recognized as significant and was included in scores 4 and 6, which yielded the highest diagnostic capabilities. In a propensity matching score study performed on 621 patients, patients taken to laparoscopic cholecystectomy with no previous ERCP were compared with patients with previous ERCP, longer surgical times and higher risk of conversion to open procedure were found in the second group^39^. In addition to this, Da Costa and Ishizaki reported preoperative ERCP as a strong predictive value for difficult cholecystectomy^28,30^.

Our recommendation in order to improve surgical outcomes is the application of a score for predicting difficult cholecystectomy; counting this study’s results into account the employment of scores 4, 6 and 7 would yield higher performances when predicting difficult cholecystectomy and are proven to be useful due to their simple implementation and widely available variables.

The 2020 WSES guidelines for the detection and management of bile duct injury during cholecystectomy recommend an exhaustive preoperative work-up prior to cholecystectomy to detect at-risk conditions, choose the best surgical approach, and discuss the risks/benefits ratio of the procedure^40^, which can be done more accurately using a scale.

Limitations in this study include its retrospective nature and the fact that it was only done in one institution. Furthermore, another limitation which was not considered was the absence of variables not included in the scores employed and it remains unbeknownst if they could have had an impact on surgical difficulty.

## Conclusions

Difficult cholecystectomies are associated with worse surgical and clinical outcomes. The standardization and use of predictive scores for difficult cholecystectomy must be implemented in order to improve surgical outcomes as a result of more meticulous planning when scheduling the procedure.

## Ethical standards

Ethical compliance with the Helsinki Declaration, current legislation on research Res. 008430-1993 and Res. 2378-2008 (Colombia) and the International Committee of Medical Journal Editors (ICMJE) were ensured under our Ethics and Research Institutional Committee (IRB) approval. Informed consent was obtained from all individual participants included in the study.

## Source of funding

This research did not receive any specific grant from funding agencies in the public, commercial, or nonprofit sectors.

## Author contribution

C.R.-G.: Study conception and design, acquisition of data, data analysis and interpretation, drafting of manuscript, critical revision of manuscript. A.I.-R.: Data analysis and interpretation, drafting of manuscript, critical revision of manuscript. D.C.M.: Analysis and interpretation of data, drafting of manuscript, critical revision of manuscript. A.C.C.-B.: Study conception and design, data acquisition, critical revision of manuscript. J.J.R.-A.: Study conception and design, data acquisition, critical revision of manuscript. I.V.-L.: Drafting of manuscript, critical revision of manuscript.

## Conflicts of interest disclosure

All authors declare no conflicts of interest.

## Research registration unique identifying number (UIN)

Researchregistry.com.

## Guarantor

Camilo Ramírez-Giraldo.

## Availability of data and material

Data are available in https://doi.org/10.34848/QSNIKJ.
